# LDL-Apheresis: Technical and Clinical Aspects

**DOI:** 10.1100/2012/314283

**Published:** 2012-04-30

**Authors:** Rolf Bambauer, Carolin Bambauer, Boris Lehmann, Reinhard Latza, Ralf Schiel

**Affiliations:** ^1^Institute for Blood Purification, Saar, 66424 Homburg, Germany; ^2^Formerly Main Hospital Darmstadt, 64283 Darmstadt, Germany; ^3^Laboratory of Medicine, 66386 St. Ingbert, Germany; ^4^Inselklinik Heringsdorf GmbH, 17424 Seeheilbad Heringsdorf, Germany

## Abstract

The prognosis of patients suffering from severe hyperlipidemia, sometimes combined with elevated lipoprotein (a) levels, and coronary heart disease refractory to diet and lipid-lowering drugs is poor. For such patients, regular treatment with low-density lipoprotein (LDL) apheresis is the therapeutic option. Today, there are five different LDL-apheresis systems available: cascade filtration or lipid filtration, immunoadsorption, heparin-induced LDL precipitation, dextran sulfate LDL adsorption, and the LDL hemoperfusion. There is a strong correlation between hyperlipidemia and atherosclerosis. Besides the elimination of other risk factors, in severe hyperlipidemia therapeutic strategies should focus on a drastic reduction of serum lipoproteins. Despite maximum conventional therapy with a combination of different kinds of lipid-lowering drugs, sometimes the goal of therapy cannot be reached. Hence, in such patients, treatment with LDL-apheresis is indicated. Technical and clinical aspects of these five different LDL-apheresis methods are shown here. There were no significant differences with respect to or concerning all cholesterols, or triglycerides observed. With respect to elevated lipoprotein (a) levels, however, the immunoadsorption method seems to be most effective. The different published data clearly demonstrate that treatment with LDL-apheresis in patients suffering from severe hyperlipidemia refractory to maximum conservative therapy is effective and safe in long-term application.

## 1. Introduction

In 1985, Brown and Goldstein were awarded the Nobel Prize for medicine for their excellent work on the regulation of cholesterol metabolism. On the basis of numerous studies, they were able to demonstrate that circulating low-density lipoprotein (LDL) is absorbed into the cell through receptor-linked endocytosis [42–44]. The absorption of LDL into the cell is specific and is mediated by a LDL receptor. In patients with familial hypercholesterolemia, this receptor is changed, and the LDL particles can no longer be recognized. Their absorption can thus no longer be mediated, leading to an accumulation of LDL in blood.

Furthermore, an excess supply of cholesterol also blocks the 3-hydrox-3 methylglutaryl-Co enzyme A (HMG CoA), reductase enzyme, which otherwise inhibits the cholesterol synthesis rate. Brown and Goldstein also determined the structure of the LDL receptor [42, 44, 45]. They discovered structural defects in this receptor in many patients with familial hypercholesterolemia [43]. Thus, familial hypercholesterolemia was the first metabolic disease that could be tracked back to the mutation of a receptor gene. Through many epidemiological studies, not only was the importance of cholesterol—and particularly that of LDL—in the development of coronary sclerosis qualitatively substantiated, but also a constant connection between cholesterol levels and coronary morbidity was established. The LDL concentration in blood is thought to be responsible for the development of arteriosclerosis and coronary heart disease, in particular [46–49].

Familial hypercholesterolemia (FH) is an autosomal dominant disorder associated with well-characterized mutations of hepatocyte apolipoprotein-B (apo-B) receptors resulting in decreased LDL removal by the liver. FH exhibits a gene dosage effect. Homozygotes may have cholesterol in the range of 650–1,000 mg/dL, xanthoma by the age of 4 years, and death from coronary heart disease by the age of 20. Heterozygotes may have cholesterol in the range of 250–550 mg/dL, xanthoma by the age of 20 years, and atherosclerosis by the age of 30 [40]. Through numerous epidemiological examinations, the importance of cholesterol—and of LDL in particular—in the development of coronary sclerosis has not only been qualitatively substantiated, but also a continuing relationship between cholesterol levels and coronary morbidity has been established [50]. The LDL concentration in the blood is particularly significant in the development of arteriosclerosis and especially of coronary heart disease.

The insight into these pathophysiological processes spurred an innovative impetus throughout both the pharmaceutical and medical industries. This innovation was aimed on one hand at metabolizing LDL intravascularly through medication or at inhibiting cholesterol synthesis and on the other hand at eliminating cholesterol from the intravascular spaces. There are various methods for the extracorporeal elimination of cholesterol, which are listed in Table 1. The standard therapy of patients with homozygous and severe heterozygous FH has been diet, lipid-lowering drugs, and LDL-apheresis. The authors will present here the different artificial extracorporeal methods for LDL-cholesterol elimination, which had influenced the prognosis of the primary and secondary dyslipoproteinemia tremendously (Table 2).

All the techniques described here are effective and well tolerated. Based on an average drop in cholesterol of 50–60 percent per session, a treatment interval of 7–14 days is advisable. The constant reduction of cholesterol is meant, above all, to prevent the progression or the development of atherosclerosis. By lowering the cholesterol from 400 mg/dL to 200 mg/dL, treatment can almost double a patient's life expectancy, according to at least one study [51]. A large disadvantage is the high costs of the different artificial methods, therefore the most LDL-apheresis treatments are performed in the industrialized nations.

## 2. Epidemiology and Pathophysiology

FH is one of the most common inherited disorders; there are 10,000,000 people with FH worldwide, mainly heterozygous. The most common FH cause is mutations along the entire gene that encode for LDL receptor protein, but it has been also described that mutations in apolipoprotein B and protein convertase subtilisin/hexin type 9 genes produce this phenotype [52]. An increased level of cholesterol is almost always due to an increase in circulating LDL, usually with simultaneous increase in VLDL and decrease in HDL. This constellation accelerates the development of arteriosclerosis and, in particular, coronary heart disease. Heterozygous familial hypercholesterolemia occurs with a frequency of 1 : 500 and the homozygous form with a frequency of 1 : 1,000,000. Patients with homozygous, familial hypercholesterolemia nearly all die before the age of 30.

Despite substantial progress in diagnosis, drug therapy and cardiosurgical procedures, atherosclerosis with myocardial infarction, stroke, and peripheral vascular disease still maintain its position at the top of morbidity and mortality statistics in industrialized nations [53]. Established risk factors widely accepted are smoking, arterial hypertension, diabetes mellitus, and central obesity. There is a strong correlation between hyperlipidemia and atherosclerosis. The role of cholesterol-bearing lipoproteins in atherogenesis is well established, and in past years the mode of interaction of these particles with cells has been elucidated. It is suggested that elevated lipid concentrations in the serum lead to their accumulation in the intima of arteries that results in the development of atherogenic plaques. These alterations seem to be accompanied by changes in vessel tone and endothelial regulation [54–57].

It has been adequately demonstrated that dyslipoproteinemia plays a key role in the pathogenesis of atherosclerosis and coronary heart disease. Previously, emphasis was placed on increase in the LDL particles. High LDL, low high-density lipoprotein (HDL), and hypertriglyceridemia affect each other. There is a strong correlation between hyperlipidemia and atherosclerosis [53, 54, 58, 59]. Various researchers have investigated the effects of hyperlipidemia on endothelial function and the relevance of these effects on early events in atherogenesis [51, 59–64]. Vascular endothelium is considered to be the largest endocrine, paracrine, and autocrine participant in the regulation of numerous homeostatic vascular functions [65]. Endothelial cells sense changes in hemodynamic forces such as pressure and shear stress as well as circulating and locally formed vasoactive substances released by blood cells. In response to these stimuli, endothelial cells synthesize and release biologically active substances such as nitride oxide (NO), prostacyclin, endothelium-derived hyperpolarizing factor, endothelins, prostaglandin H2, thromboxane A2, heparin sulfate, transforming growth factor, vascular endothelial growth factor, basic fibroblast growth factor, platelet-derived growth factor, tissue plasminogen activator, plasminogen activator inhibitor-1, oxygen-free radicals, and others [66]. These substances modulate vascular tone through their relaxing and contracting actions as well as vascular structure through production of growth-promoting and growth-inhibiting factors. In hypercholesterolemic patients, intravenous reconstituted HDL infusion rapidly normalizes endothelium-dependent vasodilation by increasing NO bioavailability. This may in part explain the protective effect of HDL on coronary heart disease and illustrates the potential therapeutic benefit of increasing HDL in patients at risk from atherosclerosis [67]. The endothelium also regulates hemostasis and thrombosis through its antiplatelet, anticoagulant, and fibrinolytic functions as well as inflammation through the expression of chemotactic and adhesion molecules [68, 69].

Endothelium plays a key role in vascular homeostasis. The endothelium is in a strategic location between the blood and vascular smooth muscle; thus it is a primary target for injury from mechanical forces and processes related to cardiovascular risk factors [65]. It is suggested that elevated lipid concentration in the serum lead to their accumulation in the intima of arteries, resulting in the development of atherogenic plaques. These alterations seem to be accompanied by changes in vessel tone and endothelium regulation [53, 56, 70]. Elevated levels of LDL increase the risk of the development and progression of coronary heart disease (CHD) [58, 65]. More recent studies have demonstrated the termination of progression, and even regression, of coronary atherosclerosis as a consequence of lipid lowering [71–73].

A positive correlation between elevated triglyceride blood levels and heart attacks has been established in numerous studies [51, 74]. Hypertriglyceridemia is prevalent in 18.6 percent of men and 4.2 percent of women between the ages of 16 and 65. Of particular importance is that increased triglycerides are often accompanied by low-HDL cholesterol blood levels. Elevated triglycerides (TG) represent a useful marker for risk of CHD, particularly when HDL levels are low [75]. The strong association between the ratio of TG/HDL and the risk of CHD suggests a metabolic interaction between the TG—and cholesterol ester-rich lipoproteins in increasing risk of CHD [76]. Dyslipoproteinemia in combination with diabetes mellitus causes a cumulative insult to the vasculature resulting in more severe disease which occurs at an earlier age in large and small vessels as well as capillaries. The most common clinical conditions resulting from this combination are myocardial infarction and lower extremity vascular disease. Ceriello et al. show an independent and cumulative effect of postprandial hypertriglyceridemia and hyperglycemia on endothelial function, suggesting oxidative stress as common mediator of such effect [77]. The combination produces greater morbidity and mortality than either alone. Early diagnosis of each condition and aggressive medical management is required to achieve an improved prognosis [78].

As an antiatherogenic factor, HDL cholesterol correlates inversely to the extent of postprandial lipemia. A high concentration of HDL is a sign that triglyceride-rich particles are quickly decomposed in the postprandial phase of lipemia. Conversely, with a low HDL concentration this decomposition is delayed. Thus, excessively high triglyceride concentrations are accompanied by very low HDL counts. This combination has also been associated with an increased risk of pancreatitis [55].

The importance of *lipoprotein (a)* (Lp(a)) as an atherogenic substance has also been recognized in recent years. Lp(a) is very similar to LDL. But it also contains Apo(a), which is very similar to plasminogen, enabling Lp(a) to bind to fibrin clots. Binding of plasminogen is prevented and fibrinolysis obstructed. Thrombi are integrated into the walls of the arteries and become plaque components. Thus, many studies show that high Lp(a) concentrations are associated with an early occurrence of coronary heart disease and apoplectic insult [79]. Due to the structural similarity between Apo(a) and a protein of the fibrinolytic system, it has not yet been possible to definitely clarify whether Lp(a) is atherogenic, thrombogenic, or both.

 Uttermann found six different Lp(a) phenotypes: S4, S3, S2, S1, B, and F. They investigated the influences of these phenotypes on the Lp(a) levels and found that phenotypes S1, S2, and B were associated with high, and phenotypes S4 and S3 with low Lp(a) concentrations [80]. It has not yet been determined whether CHD is mainly primarily associated with Lp(a) levels or with the phenotypes. It can be concluded that high levels of Lp(a) are associated with CHD; the isoforms S2, S1, B, F are linked to CHD; and patients with premature CHD showed the highest Lp(a) levels as well as the isoforms S2, S1, B, and F [81, 82]. Several mechanisms have been postulated: blocking of plasminogen, a binding site on fibrin clots, interaction with other coagulation proteins, and hepatic growth factor [83, 84]. Grainger et al. showed that Lp(a) and Apo A enhance proliferation of human smooth muscle cells in culture by inhibiting the activation of plasminogen to plasmin, thus blocking the proteolytic activation of transforming growth factor-*β* (TGF-*β*), an autocrine inhibitor of human vascular smooth muscle cells. The activation of TGF-*β* is inhibited in the aortic wall and serum of mice expressing Apo A as a consequence of Apo (a) inhibition [85, 86].

In the last years, different studies have shown that Lp(a) is a major independent risk factor for atherosclerosis, increasing cardiovascular and atherovascular morbidity and mortality at a younger age [80, 81, 87, 88]. In two large control studies in Finland and USA, Lp(a) was not associated with an increased cardiovascular risk [54, 89]. Both populations consisted of patients over 40 years of age without signs of cardiovascular disease at entry. The results of these studies indicate that high Lp(a) levels that have not led to symptomatic arteriosclerosis before the age of 40 years (and thus also led to exclusion from this study), might no longer play a role as a risk factor. After Tsimikas et al., the association of the oxidized phospholipid: apo B-100 ratio with obstructive coronary artery disease was independent of all clinical and lipid measures except one, Lp(a). However, among patients of 60 years of age or younger, the oxidized phospholipid: apo B-100 ratio remained an independent predictor of coronary artery disease [90].

Other investigators have shown that Lp(a) plays an important role in the progression of atherosclerosis. Gaubatz et al. observed that the metabolic fate of the Lp(a)-triglyceride-rich lipoprotein complex, which is more abundant in hypertriglyceridemia, may be different from that of conventional Lp(a) and may contribute uniquely to the progression or severity of cardiovascular disease [91]. Komai et al. demonstrated that oxidized Lp(a) is more potent than native Lp(a) in stimulating vascular smooth cells. Oxidized Lp(a) may play an important role in the pathogenesis of vascular disease [92]. Elevated Lp(a) levels are correlated with the extent of CHD and the mortality of these patients [93–95]. Ducas et al. reported of an acquired Lp(a) excess in patients with renal disease as a marker for cardiovascular risk [95]. The elevation of plasma Lp(a) concentrations in patients with renal diseases appears to be related to proteinuria and is, therefore, amenable to treatment. High Lp(a) levels in renal disease suggest an important role of the kidneys in the metabolism [96, 97]. Among older adults in the United States, an elevated level of Lp(a) lipoprotein is an independent predictor of stroke, death from vascular disease, and death from any cause in men, but not in women. These data support the use of Lp(a) lipoprotein levels in predicting the risk of these events in older men [98].

Thus far, no sufficiently drug therapy has been available to decrease high Lp(a) levels. N-acetylcysteine has been shown to induce a dose-dependent reduction in Lp(a) levels about seven percent by causing dissociation of the Apo A by cleavage of disulfide bonds [99]. This report has been contradicted by that of Wiklund et al. [84]. Very high Lp(a) levels can only be normalized by plasma exchange or LDL-apheresis [100].

Another strong risk factor for accelerated atherogenesis, which must be mentioned here, are the widespread high *homocysteine* levels found in dialysis patients [101]. This risk factor is independent of classic risk factors such as high cholesterol and LDL levels, smoking, hypertension, and obesity, and much more predictive of coronary events in dialysis patients than are these better-known factors. Homocysteine is a sulfur aminoacid produced in the metabolism of methionine [102]. Under normal conditions, about 50 percent of homocysteine is remethylated to methionine and the remaining via the transsulfuration pathway [103]. Vitamins are important cofactors for the enzymes in the methionine metabolism (folic acid and vitamin B12 for the remethylation pathway and vitamin B6, or pyridoxine, for the transsulfuration pathway). The kidney is an important metabolic site for removal (up to 70 percent) of plasma homocysteine [104]. In many patients, the therapy with vitamins B6 and B12 and folic acid is sufficient.

Defining hyperhomocysteinemia as levels greater than the 90th percentile of controls and elevated Lp(a) level as greater than 30 mg/dL, the frequency of the combination increased with declining renal function. Fifty-eight percent of patients with a GFR less than 10 mL/min had both hyperhomocysteinemia and elevated Lp(a) levels, and even in patients with mild renal impairment, 20 percent of patients had both risk factors present [105].

The discovery of these pathophysiological processes has led to a surge in innovation in pharmaceutics and medical technology aimed, not only at metabolization of LDL intravascular through medication or inhibition of cholesterol synthesis, but also at the elimination of cholesterol from the intravascular area with extracorporeal bioartificial methods.

## 3. LDL-Apheresis Therapy

Coronary heart disease remains one of the main causes of death in the mortality statistics of the industrial nations, despite considerable progress in diagnostics, development of new medications, such as HMG-CoA-reductase inhibitors as well as cardiosurgical measures. Cholesterol concentrations of over 200 mg/dL represent an increased coronary risk. This risk is double at cholesterol values between 200–250 mg/dL and fourfold at values of 250–300 mg/dL [106]. In addition to familial disposition, other risk factors that contribute to coronary heart disease are smoking, adiposity, diabetes mellitus, stress, reduced HDL, increased Lp(a), and fibrinogen.

Usually the severe forms of hypercholesterolemia are due to a relative or absolute reduction of LDL receptors in the liver resulting in a decreased plasma clearance of lipids [43, 44, 107]. For these patients, reduction of intake dietary fats is advised. Depending on type of condition, various medications are available, such as colestyramin, colestipol, *β*-fibrates, fenofibrate, nicotinic acid, *β*-pyridylcarbinol, probucol, and D-thyroxine. Since the introduction of HMG-CoA-reductase inhibitors, which can also be combined with other lipid-lowering drugs, LDL reduction up to 50 percent of the original concentration can be achieved. In many cases, this appears to be sufficient. Numerous studies based on large numbers of patients have investigated the effectivity and safety of the various HMG-CoA-reductase inhibitors. In these studies, investigators tested Fluvastatin, Lovastatin, Pravastatin, and Simvastatin [108–111]. During the testing period, numerous side effects like diarrhea, obstipation, other gastrointestinal diseases, myositis, rhabdomyolysis, and others were observed [112]. As a result of the clarification of the connection between hypercholesterolemia and coronary heart disease, numerous studies have been carried out in recent years with various medications [113–117]. With the introduction of selective and semiselective extracorporeal elimination methods for cholesterol, LDL, Lp(a), and triglycerides, all forms of previous therapy-resistant hypercholesterolemia can now be effectively treated [118].

Severe heterozygous forms of familial hypercholesterolemia or other forms of dyslipoproteinemia with cholesterol values between 250 and 600 mg/dL are also to be allocated to the therapy group for LDL-apheresis. Principally, these forms first require maximum dietetic and medicational therapy, for example with, 24–32 g ion exchanger in combination with 40–80 mg CSE inhibitors. If, despite this maximum therapy or due to therapy intolerance, LDL cannot be constantly held below 200 mg/dL, then LDL-apheresis is indicated. Only in cases of exceptional circumstances should patients over 60 years of age be given LDL-apheresis treatment; however, diagnosis should be supported by corresponding examinations, and the patient should be a nonsmoker. All patients should be placed under cardiological observation with ECG under exercise, thallium scintigraphy, and, possibly, coronary angiography, to register reduced progression or the desired regression of the coronary heart condition. There have been thus far not enough long-term results from controlled interventional studies with regard to the expected positive influence of LDL-apheresis implementation in coronary morbidity and mortality.

The various LDL-apheresis systems have only been part of clinical routine for the past 25 years. The advantages can, however, be estimated from the Framingham study [114, 119]. The quotient relevant for cost-effective assessment: [cost of treatment—costs saved]: [improvement in life quality] cannot be exactly calculated at present. To calculate it, detailed information is required about the expenses saved through illnesses avoided (heart attack, angina pectoris, and premature coronary death). The standard therapy for FH besides diet is, lipid-lowering drugs the LDL-apheresis. Up to now only cascade filtration immunoadsorption, heparin-induced LDL precipitation, LDL adsorption through dextran sulfate, the DALI hemoperfusion system, and the Liposorber D system have been of clinical relevance. The position of the established LDL-apheresis systems was improved by technical processes or documentation of beneficial clinical effects. For this reason, we only discuss the clinically relevant techniques here. The requirement that the original level of cholesterol is to be reduced by at least 60 percent is fulfilled by all the systems listed in Table 3.

## 4. Cascade Filtration

Cascade filtration, membrane differential filtration (MDF) or double filtration plasmapheresis seems to be superior to conventional plasmapheresis but less effective than adsorption or precipitation techniques [118–120]. The cascade filtration was developed by Agishi et al. in Japan and was the first semiselective technique used for treating hypercholesterolemia [4]. The secondary membrane in cascade filtration has a cutoff of approximately one million daltons. LDL cholesterol has a molecular weight of approximately 2,300,000 daltons and is thus retained by this membrane. All other molecules, which are larger than one million daltons are also retained, while plasma components smaller than one million daltons pass through the membrane and are returned to the patient. Thus, with plasma separation of 2,500–3,000 mL, total cholesterol can be reduced by approximately 35–50 percent of the original value and LDL cholesterol by approximately 30–45 percent thereof (Table 3) [121]. Due to irregular pore distribution with different diameters in the secondary membrane, plasma components with smaller molecular weight can also be retained, such as fibrinogen (MW: Å 340,000), HDL (MW: Å 400,000), and IgM (MW: Å 1,000,000). In the treatment with 3,000 mL of plasma not only large amounts of albumin but also other substances, as shown in Table 3 are reduced [122–125]. Given the cardiovascular risk factor, it appears beneficial to reduce fibrinogen, while decreasing HDL, a protective factor against atherosclerosis, appears to be harmful [123]. It is necessary to develop more effective secondary membranes with exactly defined pores to improve the selectivity of cascade filtration. Geiss et al. found that MDF is an effective method to lower elevated concentrations of atherogenic lipoproteins. The concomitant loss of other macromolecules transiently improves hemorrheology but demands a close monitoring of immunoglobulin concentrations as a safety parameter [126].

With the new synthetic secondary membranes such as the lipidfilter EC-50 (Asahi, Japan), and new types of machines, better effectiveness and selectivity in the separation of the blood components can be reached in the treatment of hypercholesterolemia. This cascade filtration system is called lipid filtration [127]. Klingel et al. observed during an extension period of 5 weeks with lipid filtration using the lipid filter EC-50 and treating a higher mean plasma volume of 3,370 mL, lipid filtration led to an increased reduction rate particularly for LDL cholesterol. Fibrinogen and Lp(a), with pretreatment levels of HDL cholesterol, total protein, and immunoglobulins remained unchanged and were not significantly different from the values before the last apheresis [128]. MDF or lipid filtration is as safe and effective as the HELP-system with respect to the extracorporeal removal of LDL-cholesterol, Lp(a), fibrinogen, by treating identical plasma volumes [127]. Hibino et al. observed that LDL-cholesterol removal by double filtration plasmapheresis might suppress inflammation and improve tissue environment in visceral adipose and liver tissue with fatty deposits [129].

A new term rheopheresis was created for special method of cascade filtration designed to reduce blood viscosity in the management of diseases with impaired microcirculation, like age-related macular degeneration, diabetes mellitus, coronary artery disease, peripheral arterial occlusive disease, cerebro-vascular stroke, and sudden deafness. Especially in the treatment of diabetic retinopathy and of the age-related macular degeneration, the results with the rheopheresis are very promising [130, 131]. Terai et al. observed that changes in retinal vascular diameter seem to be associated with the systemic effect of a single LDL-apheresis. Vasodilatation of the arterioles and the venules improved after LDL-apheresis, indicating an improvement of ocular perfusion in patients with hypercholesterolemia [132].

The development of new membranes with various cutoffs and improved technical apparatus will probably allow for safe and effective double or triple filtration in the near future. The implementation of specially constructed hollow fibres made of glass will also facilitate a more effective cascade filtration. These hollow glass fibres will be manufactured with pores of exactly defined measurements, which will enable exact filtration. Thus, it will be possible to manufacture various hollow fibre modules with varying pore sizes, and which are reusable. On one hand, selective plasma separation will, drastically reduce the danger of hepatitis and sensibilization by foreign proteins. The biggest advantage clinically is that physiological proteins such as clotting factors, hormones, and enzymes, and other such elements will have to a lesser extent.

## 5. Immunoadsorption

This method was first described in 1981 by Stoffel and Demant and by Borberg et al. in 1983 as LDL-apheresis with anti-LDL sepharose columns [5, 6]. Immunoadsorption means that, after primary separation, the plasma is perfused through sepharosis columns coated with LDL antibodies. The LDL molecules in the plasma are adsorbed onto the antibodies on the columns. This is a reversible antigen-antibody bond accord based on the principle of affinity chromatography. Antibodies against the protein component in human LDL cholesterol (apolipoprotein B 100) gained from sheep are covalently bound to sepharose particles. These are heteroclonal sheep antibodies against apoprotein B, which are bound to sepharose after cyanogen bromide activation. In one column, three grams of LDL cholesterol can be adsorbed. Both columns contain 300–320 mL of sepharose particles.

Before the column is saturated with the absorbed lipoproteins (600–800 mL plasma), the plasma flow is switched to the other column; while one column is used for adsorption, the off-line column is generated with neutral saline buffer solution, glycine buffer (pH 2.4), and neutral buffer again. The treated plasma is then mixed with the cellular components of the blood and returned to the patient. The entire procedure takes 2.5–3 hours via a computerized apheresis monitor. After the treatment, the columns are rinsed, and after the same procedure, filled with sterile solution. The immunoadsorption columns can be used for a minimum of 40 treatments.

The antigen-antibody bound is reversed by using a mixture of glycine and hydrochloric acid with a pH of 2.8. The pH is then increased to 7.4 using a sodium chloride solution, buffered with phosphate, and rinsed with physiological common salt solution. This procedure restores the binding capacity of the columns and prepares them for use again. In this way, any required volume of plasma can be perfused in one treatment session (3–10 L). Upon conclusion of the treatment, the columns are regenerated, sterilized, and can be implemented again. The advantage of this method is the selectivity, effectiveness, and regenerating capacity of the columns (Table 1). The disadvantage is the high expenditure required not only for the treatment itself, but also for the regeneration process. The immunoadsorption columns are approved for regular and continued use [133].

Given the high cost of the columns, implementation of this system is only viable on a long-term basis, that is to say, at least 40 times per patient. At a perfusion volume of 3–6 liters per session, the LDL cholesterol is reduced to 30–40 percent of the original level. HDL, serum proteins, immunoglobulins, and fibrinogen, and so forth drop by approximately 15–20 percent and return to their normal level, however, after approximately 24 hours. Parusel et al. reported findings of a temporary activation of the complement system and a drop in leucocytes [134]. Resulting side effects or other complications have, only been seldom reported, however [13].

Two different systems are currently available, the LDL-Therasorb system (Miltenyi Biotec, Germany) and the LDL- and Lp(a)-Excorim system (Fresenius, Germany). The matrix sepharose is coupled with specific antihuman apolipoprotein B-100 or antihuman Lp(a) sheep antibodies. Both systems are safe and effective in clinical use, even in long-term treatment. Indications for the extracorporeal elimination of LDL cholesterol are primary and secondary dyslipoproteinemia, and for the Lp(a) IA the solitary familial Lp(a) elevation.  Table 4 shows a compilation of some of the results of IA. Jovin et al. found no significant variation of selectivity. The efficacy of the LDL-apheresis immunoadsorption columns did not decrease after 60 treatments sessions. The columns selectivity also remained unchanged [135]. The advantages of immunoadsorption are high selectivity and effectiveness for adsorption of all apo-B-containing lipoproteins. This treatment showed a beneficial effect of long-term LDL-apheresis on atherosclerotic vascular disease [15, 136, 137].

## 6. Lipoprotein (a)-Apheresis

Lipoprotein (a) (Lp(a)) represents a class of lipoprotein particles which have lipid composition similar to LDL and a protein moiety, apo-B 100, covalently linked to apo(a), a glycoprotein with striking structural similarities to plasminogen [138]. High plasma levels of Lp(a) are associated with an increased risk for atherosclerotic coronary heart disease (CHD) by a mechanism yet to be determined [139]. Because of its structural properties, Lp(a) can have both atherogenic and thrombogenic potentials [81]. The means for correcting the high plasma levels of Lp(a) are still limited in effectiveness. All drug therapies tried thus far have failed. The most effective therapeutic methods in lowering Lp(a) are the LDL-apheresis methods. Since 1993, special immunoadsorption polyclonal antibody columns (Pocard, Moscow, Russia) containing sepharose bound anti-Lp(a) have been available for the treatment of patients with elevated Lp(a) serum concentrations [100, 140].

Monospecific polyclonal antibody to human Lp(a) was obtained from immune sheep serum. Pokrovsky et al. described preparing immunosorbent by immobilizing of antibodies to Sepharose CL-4B [140]. For the treatment, two columns are necessary. Each column is filled with 400 mL of sorbent tested for sterility and pyrogenicity. Anti-Lp(a) immunoadsorption columns are reusable. Between the treatments, the columns are stored at 4°C in storage solution, which is rinsed prior to each Lp(a)-apheresis procedure. Two personal columns are assigned to each patient [141].

The numerous studies provide the epidemiological evidence that Lp(a) is an independent risk factor in the pathogenesis of coronary heart disease and arteriosclerosis. The individual plasma level is genetically determined. Diet or available drugs do not influence the plasma level of Lp(a). Until today there is only one device available on the market which allows the specific removal of Lp(a) from plasma (Lipopak, Pocard, Moscow, Russia). As with LDL-apheresis in homozygotes-specific Lp(a) is a life-saving therapy in severe cases with elevated Lp(a) as the sole risk factor.

## 7. Heparin-Induced LDL Precipitation (HELP)

In 1982, Seidel and Wieland reported on a new method of extracorporeal elimination of low-density lipoproteins [142]. This method was abbreviated HELP. After primary separation, the plasma is mixed in a ratio of 1 : 1 with an acetate-acetic acid buffer (pH 4.85), so that the pH of this mixture is 5.1. Then, 100,000 U heparin per liter are added to the buffer. After the plasma has been mixed thoroughly with the acetate-acetic acid buffer and heparin, LDL cholesterol precipitates in the acid environment together with fibrinogen and heparin to form insoluble precipitates. These precipitates are then removed from the plasma by means of a polycarbonate membrane. The remaining free heparin is almost completely removed by a heparin absorber (DEAE cellulose). The acidulous plasma is returned to a physiological pH value using bicarbonate dialysis, and the plasma, free of LDL, is returned to the patient with the blood cells [143]. Although the method is technically complicated, it is reliable and effective. It is, however, nonselective for in addition to the cholesterols, C3, C4, fibrinogen, plasminogen, and factor VIII, and so forth are also eliminated (Table 3). HDL reaches its original level after 24 hours, while the fibrinogen concentration only increases gradually; the amount of plasma should, therefore, be limited to 3 three litres [143].

More recently, a compact unit has been designed that somewhat reduces the cost of the equipment. The Plasmat Futura (B. Braun, Germany) is easy to use and safe in handling. The priming rinsing and reinfusion are fully automated. The entire treatment uses only disposable material, the machine does not need descaling or disinfection, and no piped water supply or reverse osmosis is required. The user is safely guided through all treatment steps and supported with message prompts and warnings.

Because C3, C4, fibrinogen, and plasminogen are also reduced to approximately 50 percent of their original concentration as well as cholesterol, LDL and triglycerides, the technique is not highly specific [143]. Due to the large drop in fibrinogen in the HELP process, the amount of plasma per session is limited to 3 three liters. A treatment using larger amounts of plasma could lead to bleeding complications. With this amount of plasma, the cholesterols are reduced as shown in Table 3. The other factors, like CRP, are also reduced by approximately 50 percent. In contrast, HDL is only reduced by 10–20 percent. Thus far no severe side effects have been described; however, occasional shivering and drops in blood pressure have been observed [22].

Studies on patients undergoing regular extracorporeal LDL-elimination indicate that the incidence of adverse cardiovascular clinical events can be reduced much earlier than by drug therapy alone. These immediate clinical benefits, which take place directly after the apheresis, cannot be due, however, to an improvement in coronary morphology (i.e., regression of atherosclerotic lesions), since such improvement can only be observed after several months of treatments [22]. The improved myocardial perfusion and clinical symptoms after LDL-apheresis is likely due to an improvement in rheology as well as producing an immediate positive influence on endothelium function and an increase in the volume of vasodilatory, antiaggressive nitrogen monoxide released or a reduction in the volume of available endothelin [26, 144]. A more than 60 percent reduction of LDL at weekly intervals is clearly associated with an early regression of lipid-rich vascular lesions [145]. LDL apheresis reduces the shear-stress of the flowing blood on vulnerable plaques either by its effect on plasma viscosity and/or on the vasomotoric reserve, thus leading to a lower peripheral arterial resistance. Furthermore, LDL-apheresis eliminates oxidized LDL, which might counteract plaque stabilization by its inflammatory effects. LDL-apheresis to with coagulation factors normalizes hypercoagulatory states, thus preventing atherothrombotic events at the site of vulnerable or erosive plaques [145].

The safety and long-term applicability of the HELP system has been proved in more than 120,000 treatments. Serious complications have never been observed [20], and the technology of the equipment has been improved over time. Many authors have shown that there is clear clinical evidence that a drastic lowering of LDL concentrations by HELP reduces significantly the rate of total and coronary mortality as well as the incidence of cardiovascular events in high-risk hypercholesterolemic patients [20, 23, 144, 146, 147]. Wang suggested that simultaneous reduction of proinflammatory and prothrombotic factors with atherogenic lipoproteins by HELP apheresis may contribute to improvement of endothelial dysfunction and thereby inhibit progression of atherosclerotic lesions and stabilize the existing plague [28]. Otto et al. found that LDL apheresis slightly, but significantly reduced CRP concentrations in patients with CHD on statin therapy, which may contribute to the stabilization of atherosclerosis in hypercholesterolemic patients treated with LDL apheresis [148]. These results are even more impressive when known age-related increase in CRP over treatment period is taken into account [148, 149].

All HELP treatments have demonstrated successful secondary prevention for patients with familial hypercholesterolemia, coronary artery disease, cardiac bypass, or heart transplantation [23, 150, 151]. Elimination of fibrinogen and other substances also has an influence on blood viscosity, rheology, and erythrocyte aggregation; thus, the microcirculatory situation as a whole can be significantly improved. Numerous reports on clinical results with the HELP technique have been published in recent years [18, 146, 152]. A selection of literature and LDL elimination has been compiled in Table 5. Severe side effects are rare: so far, the only side effects reported have been transient shivering and hypotension. More than 83 percent of the treated patients have demonstrated clinical improvement.

In 1999, Jaeger et al. published the first clinical results with HELP apheresis in acute cerebral infarction (stroke). These results are similar to the clinical experience reported with myocardial ischemia [150]. In the same year, Suckfüll et al. published a randomized study of the clinical utility of LDL apheresis in the treatment of sudden hearing loss [153]. Their results suggest that the clinical outcome of the treatment of sudden hearing loss after a single HELP apheresis is superior to the more expensive treatment with prednisolone, dextrans, and pentoxifylline. Bianchin et al. found that in a specific group of patients with alterations in cholesterol and/or fibrinogen, the HELP apheresis treatment is a further option available in sudden sensorineural hearing loss [154]. More studies should be needed to show whether or not the HELP apheresis will be the most effective treatment for these diseases.

## 8. Dextran Sulfate Low-Density Lipoprotein (Liposorber)

Mabuchi et al. reported performing a study in 1987 on LDL absorption with dextran sulphate (Liposorber LA-15, Kaneka, Japan) [10]. Low-molecular dextran sulfate (MW 4500) can selectively absorb all substances containing apolipoprotein B. Dextran sulfate is covalently bound to cellulose particles. The dextran sulfate was selected as an affinity ligand of LDL adsorbent for its high affinity and low toxicity. The binding mechanism is the direct interaction between dextran sulfate and the positively charged surface of apolipoprotein B-containing lipoproteins (LDL, VLDL, and Lp(a)). The dextran sulfate has a structure similar to that of the LDL receptor and seems to act as a type of pseudoreceptor [34, 155–157]. Approximately 2.5 grams LDL can be bound per column. After primary separation, the plasma is perfundated through the columns, where all material containing Apo-B such as cholesterol, LDL, VLDL, and triglycerides is absorbed. Free of cholesterol, the plasma is returned to the patient. After 500 mL of plasma, the columns are saturated and require regeneration with 4.1 percent NaCl solution [155]. After rinsing with Ringer's solution, they are ready for use again. The effectiveness of this treatment is good, and cholesterol is eliminated selectively (Table 1). Occasionally, with a perfusion volume of more than four liters, a marked drop in the Quick level can occur, probably caused by the absorption of factor VIII [34].

This method has also found widespread clinical use in recent years [156–161]. Some of the reported LDL reduction rates have been compiled in Table 6. Side effects are rare and of a minor nature such as hypotension, nausea, hypoglycemia, and light allergic reactions. In a study we conducted with 20 patients and 955 LDL-apheresis sessions, we observed that these side effects were observed in 12 percent of all treatment sessions. A shock situation only arose in 0.4 percent, and in 0.2 percent allergical reactions occurred, which were easily treated [16]. The dextran-induced allergical reactions have not been observed so far. Low-molecular dextran sulfate is much less allergenic than the forms of dextran which are normally implemented as a plasma expander and have a higher molecular weight of 40,000 to 80,000. The advantage of the Liposorber system is the selectivity by elimination of all apo B-containing lipoproteins and the high effectiveness. A disadvantage is the labor intensive technology.

More than 60 percent reduction of the pretreatment cholesterol values can be achieved by one treatment with the Liposorber system. The effectiveness of therapy has also been observed over time in several long-term clinical studies (Table 6). In more than 75 percent of cases, patients improved or reached regression of coronary atherosclerosis. The observed side effects were between 0.5 and 4 percent [30, 31, 37, 53, 155]. The Liposorber system is safe and effective, even in high-risk hypercholesterolemia patients. Although evaluation of the effectiveness of LDL-apheresis on coronary arterial lesions has not yet been fully established, evidence is accumulating to show, not only that it prevents the development of coronary heart disease, but also can effect its regression by lowering of cholesterol to an optimum level in all untreatable hypercholesterolemia patients [53].

In 1994, Kroon et al. presented data on a pregnancy in a 20-year-old woman with homozygous familial hypercholesterolemia and coronary heart disease who was treated with the Liposorber LA-15 system biweekly [162]. During pregnancy and delivery, no signs of maternal coronary insufficiency developed. Serial ultrasonic measurements of fetal growth indices and the blood flow velocity waveforms of the urine and umbilical artery revealed no sign of fetal growth retardation or insufficiency of the uteroplacental circulation. Even in children, the Liposorber system has proved to be safe and effective. Stefanutti et al. reported in 1997 of a 4.5-year-old-girl with homozygous familial hypercholesterolemia and CHD who was treated effectively with LDL apheresis [163]. The decrease of the atherogenic apolipoprotein B-containing lipoproteins was between 50 and 70 percent. The child tolerated the LDL-apheresis without any clinically significant complications. She was submitted to a long-term program of treatment at biweekly intervals. The experience of these authors led them to recommend early therapeutic intervention with extracorporeal treatment with LDL-apheresis in children severely affected by homozygous or double heterozygous familial hypercholesterolemia.

Two clinical reports described excellent long-term follow-up results for patients with coronary artery disease who had been treated with LDL-apheresis using dextran sulfate cellulose columns plus adjunctive cholesterol lowering drug therapy [164]. In addition, there is increasing evidence that LDL-apheresis is effective for the prevention of extracoronary atherosclerotic disease, and it is also reported to have the potential to improve microvascular disorders. Since the mechanisms of clinical improvement caused by LDL apheresis extends beyond simple and drastic reduction of LDL cholesterol, further investigation based on recent vascular biological evidence is needed [165]. Intensive cholesterol-lowering therapy with LDL-apheresis and lipid-lowering drugs can achieve a substantial decrease in LDL-cholesterol levels to induce regression of coronary artery disease [32]. The Liposorber LA-15 (Kaneka, Japan) system represents a safe and effective therapy option for patients suffering from severe hypercholesterolemia that could not be adequately controlled by diet and maximum drug therapy.

Complement activation takes place with essentially all adsorbers; however, activated complement is removed by binding to specific proteins such as C4 binding protein. In the presence of anticoagulating heparin, bradykinin is formed, but the production of bradykinin is low and normally not clinically significant [166, 167]. However, drugs that inhibit angiotensin converting enzyme (ACE inhibitors) can exacerbate the clinical effect of this amount of bradykinin to the point of serious adverse effect including anaphylaxis [168, 169].

Olbricht et al. reported on a anaphylactic reaction in a patient during ACE inhibition therapy and LDL-apheresis [169]. He observed reactions similar to those which frequently occur in ACE inhibition therapy in combination with AN-G9-high-flux dialysis as well as with reusable polysulphone membranes in intermittent hemodialysis [170, 171]. The functioning mechanism of these anaphylactic reactions has still not been finally clarified, although they are presumably induced by the increased release of bradykinin. Bradykinin is formed through activation of the contact activation system, which consists of the components: high-molecular-weight kininogen, prekallikrein, Hagemann's factor, and coagulating factor XI. When plasma comes into contact with very high-negatively charged dextran sulfate, the concentration of bradykinin in the plasma increases considerably [170, 171]. Bradykinin is quickly decomposed through the activity of kininase I and II; thus patients undergoing lipid apheresis are not normally affected.

Kininase II is identical to angiotensin-converting enzyme and is blocked by the administration of ACE inhibitors. This results in an increase of bradykinin in the bloodstream and to an anaphylactic reaction. These anaphylactic reactions are not specific to a particular membrane or surface type, but can always occur in ACE inhibition when blood or plasma comes into contact with contact-activating surfaces. The reactions are independent of the type of ACE inhibitor; the presence of dialysate is not necessary, for they also develop in cell-free plasma. The varying degrees of severity are presumably connected to the expression of the ACE-coding gene being subject to strong individual fluctuations [172]. In the instructions of the LDL apheresis procedure, there is a contraindication for ACE inhibitors by LDL-apheresis. ACE inhibitors must not be administered to patients undergoing LDL-apheresis [173].

## 9. Low-Density Lipoprotein Hemoperfusion

### 9.1. DALI System

 In 1993, Bosch et al. first described low-density lipoprotein hemoperfusion (direct adsorption of lipoproteins (DALI), Fresenius, Germany) [174]. The new adsorber, which is compatible with human whole blood, uses a matrix of polyacrylate beads. In the DALI system, blood is perfused through the adsorber, which contains 480 mL of polyacrylate-coated polyacrylamide, without regeneration. The column has a capacity of more than 1.5–2.0 blood volumes for effective adsorption of cholesterol, LDL, Lp(a), and triglycerides. Regeneration is not necessary because the column is used for only one treatment [175, 176]. In a very simple extracorporeal circuit, the blood is pumped through the LDL adsorber. The elimination of LDL, Lp(a) particles, and other lipoproteins from whole blood is performed by adsorption onto polyacrylate-coated polyacrylamide beads [177]. The small porous beads with a diameter of 150–200 *μ*m are immobilized in the adsorber with the aid of two sieves. The beads have a porous structure which exploits the principle of size exclusion chromatography. The sponge-like structure of the beads offers a very large inner and outer surface for adsorption (more than 99 percent of the overall surface of over 1,000 m^2^ is located within the beads).

The adsorption of LDL and Lp(a) and other lipoproteins occurs by polyacrylate ligands covalently binding to the polyacrylamide surface. Like the LDL-receptor, polyacrylate, consists of polyanions, with negatively charged carboxylate groups [175]. The polyanions interacts selectively with the cationic groups in the apoprotein B moiety of LDL and Lp(a). Due to this electrochemical interaction, the lipoproteins are immobilized on the beads. By flowing, the whole blood past the beads affects only a minor interaction between the blood cells and the similarly small outer surface of the beads [177, 178]. The smaller lipoproteins can easily penetrate the inner sponge-like structure of the beads via the pores. HDL can also penetrate the beads, but because the apo AI-coated HDL is not attracted to the ligand, it is not affected by the adsorber and cannot be eliminated. Monitoring of this simple extracorporeal blood circulation system is carried out by measuring the blood pressure in the afferent and efferent blood lines and at the adsorber inlet. Anticoagulation is carried out by first applying a heparin bolus, then by a continuous ACD-A solution infusion into the blood line as it exits the patient's vein. In most cases, an infusion rate of one mL of acid citrate dextrose per 20–25 mL of blood is sufficient for adequate anticoagulation of the extracorporeal circuit. The speed of the ACD-A pump controls the amount of solution, which is monitored by a drip counter.

Besides lipoproteins, the DALI system also adsorbs the positively charged ions calcium and magnesium. Therefore, the columns have to be prerinsed with 4–6 liters of a priming solution containing these electrolytes. The adsorber is thereby saturated with these cations, thus preventing hypocalcemia and hypomagnesia during the treatment [178]. The DALI system can be run at three different adsorber sizes (DALI 500, 750, and 1000 mL adsorbers). Special equipment and tubes are available. After passing the adsorber, the blood depleted of all apoB-containing lipoproteins is put back into the patient. The advantages are good selectivity, high effectiveness, and a simple technology. The potential for possible microparticle release from the columns as with all adsorbers can be avoided prevented by more and better rinsing of the columns and a careful handling. A second leucocyte type in line filter can be added after column to further reduce the possibility of microparticle release. Such a filter is recommended to maximize safety. In a five-year followup, long-term therapy with DALI was safe, effective, and selective as LDL and Lp(a) could be reduced by >60 percent per session in approximately 100 minutes treatment time, while decrease and the incidence of side effects were low [179]. The DALI system has proven to be safe, effective, and simple to perform.

### 9.2. Liposorber D

 Another whole blood lipoprotein apheresis system (Liposorber D, Kaneka Japan) is commercially available too. The Liposorber D system is the second whole blood perfusion type LDL apheresis system developed on the basis of the technology of the dextran sulfate Liposorber LA-15 system. Liposorber D adsorbs positively charged LDL, VLDL, and Lp(a) particles from whole blood using negatively charged polyanions. Liposorber D contains negatively charged dextran sulfate covalently bound to cellulose [12]. The negatively charged surfaces activate the intrinsic coagulation pathway; prolongation of a PTT and shortening of PT have been observed in the LDL apheresis with the dextran sulfate column. Coagulation factors such as factors XI and XII were reduced by dextran sulfate adsorption, but those coagulation factors returned to normal range within one or two days after the treatment. A Japanese multicenter clinical trial found a significantly reduce in LDL, Lp(a), and triglycerides by using Liposorber D [180]. Adverse events, which were observed, were hypocalcemia during treatment caused by ACD-A solutions, the symptoms disappeared by administration of calcium, and slight hypotension.

From a technical point of view, the Liposorber D whole blood adsorption column has clear advantages over the usual LDL-apheresis systems that require plasma separation. The system is simpler and easier to handle because no plasma separation procedure is necessary. In vitro evaluations have shown that the adsorbent efficiently adsorbs LDL and Lp(a) with a good biocompatibility. The clinical results of this technology are very encouraging. The advantages are good selectivity, effectiveness, and a simple technology [12]. Possible columns configurations DL 50, DL 75, and DL 100 are available.

A treatment performed with the Liposorber D and the new developed machine DX-21 reduces the apoB lipoproteins without having great influence on HDL-C, other important plasma components or blood cells. Liposorber D chemically is identical to that for Liposorber LA-15 with a modification of the size of the adsorbent beads suitable for whole blood processing. The Liposorber D system is comparable with the DALI system. The handling with the DX-21 machine is easy, safe, and the user is guided through all treatment steps.

In conclusion, the developed whole blood perfusion LDL-apheresis system Liposorber D is a safe and simple apheresis system and thus a useful modality to remove LDL-C and Lp(a) from whole blood in hypercholesterolemic patients.

- Liposorber D is especially designed to eliminate LDL-C and Lp(a) from the whole blood.
- The adsorbent for Liposorber D, dextran sulfate-cellulose, is chemically identical to that for Liposorber LA-15, with a modification of the size of the adsorbent beads suitable for the whole blood processing.
- The whole blood processing without plasma separation provides a simple, easy-handling, and a shorter operation time.

## 10. Conclusion of LDL-Apheresis

All of the techniques described above are effective and well tolerated. With weekly or biweekly treatment, the average LDL cholesterol concentration can be reduced to approximately 50–60 percent of the original levels. LDL concentration increases again after each apheresis session, but does not return to the original level. After a few sessions, it balances out. The increase after apheresis can be slowed down by lipid-lowering drugs. By lowering the cholesterol from 400 mg/dL to 200 mg/dL, treatment can almost double a patient's life expectancy. In Tables 4–6, results are only given for the presented observation phase. The LDL-apheresis treatment must be repeated after the above-mentioned treatment procedure in homozygous and severe heterozygous FH life-long or until other better therapy technologies are available.

LDL-apheresis decreases not only LDL mass but also improves the patient's life expectancy. LDL-apheresis performed with different techniques decreases the susceptibility of LDL to oxidation. This decrease may be related to a temporary mass imbalance between freshly produced and older LDL particles. Furthermore, the baseline fatty acid pattern influences pretreatment and postreatment susceptibility to oxidation [25].

Streicher et al. observed that despite drug therapy, LDL-apheresis significantly stimulates the residual LDL-receptor expression in FH via the reduction of available extracellular cholesterol resulting in delayed reappearance of hypercholesterolemia in between treatments [181]. The acute effect of lipid apheresis on serum lipidome could be predominantly attributed to lipoprotein changes, while blood cell damages during this procedure caused additional, less-pronounced changes. The importance of specific changes in particular lipid species remains to be established [182].

The techniques vary somewhat in selectivity. Cascade filtration reduces HDL concentration, which probably has an atherogenous effect in the long term. HELP, cascade filtration, and dextran sulfate adsorption to a lesser extent, and whole blood hemoperfusion systems reduce the average fibrinogen concentration. This reduction can prove advantageous as the viscosity of the blood is reduced and the rheological characteristics improved. Moreover, fibrinogen is an independent risk factor in the development of coronary heart disease.

The primary aim in reducing cholesterol concentration is to prevent the development and progression of atherosclerosis. There are sufficient data that this therapeutic aim can be achieved. There are many reports on decrease and slower progression of atherosclerotic changes in coronary vessels and carotids after patients have been treated for one or more years with lipid apheresis. Selective LDL elimination through LDL-apheresis represents a decisive breakthrough in the treatment of high-risk patients with hypercholesterolemia, whose treatment has, up to now, been inadequate, despite strict diets and lipid-reducing medication.

The German Federal Committee of Physicians and Health Insurance Funds criteria for LDL-apheresis are

1. FH homozygotes,
2. patients with severe hypercholesterolemia in whom maximal dietary and drug therapy for >1 year have failed to lower cholesterol sufficiently [183].

The Apheresis Applications Committee (AAC) of the ASFA summarized the LDL-apheresis in familial hypercholesterolemia as follows. The goal of LDL-apheresis is to reduce the time-averaged total cholesterol levels by 45–55 percent, the LDL levels by 40–60 percent, and the Lp(a) by 40–60 percent [40]. FDA approved indications for patients with FH unresponsive to pharmacologic and dietary management are

1. functional homozygotes with a LDL cholesterol >500 mg/dL,
2. functional heterozygotes with no known cardiovascular disease but a LDL cholesterol >300 mg/dL,
3. functional heterozygotes with known cardiovascular disease and LDL cholesterol >200 mg/dL [40].

Patients without FH but with high LDL or Lp(a) cholesterol who cannot tolerate or whose conditions are unresponsive to conventional therapy can also be treated [40]. During pregnancy, LDL cholesterol levels in individuals affected by FH can rise to extreme levels that can compromise uteroplacental perfusion. There are case reports of the use of LDL-apheresis in this indications to allow for successful completion of pregnancy. TPE can be effective but because of the availability of the selective removal systems and their enhanced efficiency of cholesterol removal, the use of TPE to treat FH is uncommon. It may, however, be the only option in small children [40]. The guidelines on the use of TA in hyperlipidemia are shown in Table 7.

In 2010, the AAC has concerted effort to generate a system, which better translates the existing evidence to treatment of the individual patient. The AAC introduced the grading recommendations 1A, 1B, 1C, 2A, 2B, and 2C including strong recommendation (1A) to weak recommendation (2C) [41].

A reduction in costs is a valid demand in view of the scarce resources available in the healthcare system. Commissions, consisting of physicians, administration specialists and representatives of the health insurance funds and others, nowadays decide at a “round table” who will be granted medical facilities and who will not, this is a clinical routine adopted only in Germany. Physicians are committed to helping all the patients entrusted to them to the best of their knowledge, and this means that medical treatment—and particularly the apheresis processes—must become affordable. This demand represents a great challenge to physicians, politicians, health organisations, and, above all, to the manufacturers. Industry constantly justifies the high costs with the extensive research and development required. All those involved in the healthcare system must intensify their cooperation in this respect.

Nevertheless, medical progress is advancing and will not be stopped. Since the introduction of hollow fiber membranes, exceptional efforts in research and development have been undertaken in the apheresis sector alone, enabling, for example, the introduction of selective separation techniques into everyday clinical practice—techniques which were unthought of—at the beginning of the eighties. This is reflected in the numerous national and international specialist congresses, which take place each year.

## Figures and Tables

**Table 1:** Extracorporeal methods for elimination of LDL cholesterol [1].

Year	Authors	Method	Advantage	Disadvantage
1967	De Gennes et al. [2]	Plasmapheresis	Quick and well-tolerated elimination of pathologic substances	Unselectivity, danger of infection, bleeding, and risks of human albumin
1975	Thompson et al. [3]	Plasmapheresis		
1980	Agishi et al. [4]	Cascade filtration	Semiselectivity	Danger of infection and low effectiveness
1981	Stoffel and Demant [5]	Immunoadsorption	Selectivity, effectiveness, regeneration, and reusability	Expensive technology
1983	Borberg et al. [6]	Immunoadsorption		
1983	Wieland and Seidel [7]	Heparin-induced LDL precipitation (HELP)	Selectivity and effectiveness	Expensive technology
1985	Nose et al. [8]	Thermofiltration	Selectivity and effectiveness	Outdated technology, behavior of macromolecules under heat unknown and not available
1985	Antwiller et al. [9]	Dextransulfate-induced LDL precipitation	Selectivity and effectiveness	Expensive technology and not available
1987	Mabuchi et al. [10]	Dextransulfate LDL adsorption (liposorber-LA 15)	Selectivity and effectiveness	Expensive technology
1993	Bosch et al. [11]	LDL hemoperfusion (DALI)	Selectivity, effectiveness, and simple technology	Unknown
2003	Otto et al. [12]	LDL hemoperfusion (liposorber D)	Selectivity, effectiveness, and simple technology	Unknown

**Table 2:** Possible indications for extracorporeal elimination of Cholesterols [1].

Dyslipoproteinemia	
Primary	Secondary
Familial hypercholesterolemia	Endocrinological diseases
Familial apo-100 defect	Diabetes mellitus
Polygenetic hypercholesterolemia	Nephrotic syndrome
Familial combined hypercholesterolemia	Renal-, heart transplantation
Isolated lipoprotein (a) elevation	Hemodialysis

**Table 3:** Effectiveness of the various LDL techniques (reduction in percent of original concentration, (according to [1]).

	Cascade filtration (2,500–3,000 mL plasma) reduction of original concentration (%)	Immunoadsorption (4,000–5,000 mL) (%)	Heparin-induced LDL precipitation (HELP) (2,500–3,000 mL) (%)	LDL-adsorption (dextran sulphate) liposorber (2,500–3,000 mL) (%)	LDL hemoperfusion (DALI) 1.6 blood volume (%)	LDL-hemoperfusion (liposorber D) 1.5 blood volume (%)
Cholesterol	35–50	30	50	45	60	55
LDL	30–45	35	45	35–40	60–75	60–75
HDL	35–50	20	10–20	—	16–29	5–13
Lp(A)	60–70	60	46	60	60–75	60–75
Triglycerides	60	60	60	70	ca 40	ca 66
Fibrinogen	50	10–20	50	30	16	20–40
IgM	35	10–20	—	—	21	14
IgA	55	10–20	—	—	—	—
Factor VIII	—	10–20	10–20	20	—	—
C 3	—	—	50	—	—	—
C 4	—	—	50	—	—	—
Plasminogen	—	—	50	—	—	—

**Table 4:** Immunoadsorption (Therasorb, Miltenyi, Germany) in hypercholesterolemia (selection of literature).

Year	Authors	Patients (*n*)	Duration of therapy (years)	Reduction of LDL (%)
1988	Borberg et al. [13]	5	3–5	52–67
1988	Oette and Borberg [14]	10	5–8	71
1990	Richter et al. [15]	8	1–3	56
1993	Bambauer et al. [16]	4	1-2	55–58
1996	Richter et al. [17]	18	8.6	60–70

**Table 5:** Clinical results with the HELP-LDL apheresis system (B. Braun, Germany) (literature overview).

Year	Authors	Diagnosis	HELP patients (*n*)	Drop out	Therapy duration (years)	Side effects (%)	Outcome
1987	Eisenhauer et al. [18]	FH, CHD	13	—	0.5–1.3	?	13 improved
1988	Thiery et al. [19]	FH, CHD	7	—	0.5–1.0	3.2	7 improved
1991	Seidel et al. [20]	FH, CHD	51	5	1.0	2.9	46 improved
1993	Bosch et al. [21]	FH, CHD ESRD	3 (HD)	—	1.5	13.0	3 improved
1994	Schuff-Werner et al. [22]	FH, CHD	51	12	2.0	2.8	39 improved
1997	Jaeger et al. [23]	FH, CHD HTX	15	—	3.6	—	5 improved
1998	Mellwig et al. [24]	FH, CHD	9	—	?	—	8 improved
1999	Donner et al. [25]	FH	4	—	?	?	4 improved
2000	Schettler et al. [26]	FH, CHD	18	—	>0.5	—	18 improved
2001	Moriarty et al. [27]	FH	4	—	0.5	—	4 improved
2008	Wang [28]	FH, CHD	22	—	1.0	—	22 improved

ESRD: end-stage renal disease and HTX: heart transplantation.

**Table 6:** LDL adsorption with dextran sulfate (Liposorber LA-15, Kaneka, Japan) in hypercholesterolemia (selection of literature).

Year	Authors	Diagnosis	Patients (*n*)	Drop out	Therapy duration (years)	Side effects (%)	Outcome
1988	Thompson and Okabayashi [29]	FH, CHD	20	—	2.1	0.5	19 improved
1992	Gordon et al. [30]	FH	54	—	0.25	—	54 improved
1994	Daida et al. [31]	FH, CHD	66	—	1.0	—	45 improved
1996	Kroon et al. [32]	FH, CHD	21	—	2.0	1.3	21 improved
1997	Gordon and Saal [33]	FH, CHD	45	4	0.5	4.0	41 improved
	Bambauer et al. [34]	FH, CHD	120	35	6.0	2.2	85 improved
1998	Mabuchi et al. [35]	FH, CHD	130	—	6.0	—	94 improved
1999	Nishimura et al. [36]	FH	30	5	2.3	—	4 improved
	Richter et al. [37]	FH, CHD	8	—	3.0–5.0	—	6 improved
2003	Bambauer et al. [38]	FH, CHD	32	—	8.0	0.3	31 improved
2010	Moriarty et al. [39]	FH, CHD	10	—	0.5	—	10 improved

**Table 7:** Guidelines on the use of TA in hyperlipidemia [40, 41].

	Apheresis applications committee of ASFA			
	2007		2010	
	TA modality	Category	Category	Recommendations grade
FH, homozygous	Selective removal methods TPE	** I**	** I** **II**	** 1A** ** 1C**
FH, heterozygous LDL > 300 mg/dL	Selective removal methods TPE	** II**	** II**	** 1A ** ** 1C**
FH heterozygous with CDH	Selective removal methods TPE	** II**	** II**	— —
FH during pregnancy	Selective removal methods TPE	** II**	** II**	— —
CHD and elevated Lp(a) > 60 mg/dL	—	—	—	—
Hypertriglyceridemic pancreatitis (chylomicronemic syndrome)	TPE	** III**	** III**	** 2C**
